# Nationwide Prevalence of Dental Caries in Turkish Children: A Meta-Analysis

**DOI:** 10.3390/children12060777

**Published:** 2025-06-14

**Authors:** Ezgi Eroğlu Çakmakoğlu, Ayşe Günay

**Affiliations:** 1 Department of Pediatric Dentistry, Faculty of Dentistry, Adiyaman University, Adiyaman 02000, Turkey; 2 Department of Pediatric Dentistry, Faculty of Dentistry, Dicle University, Diyarbakir 21000, Turkey; ayok18@hotmail.com

**Keywords:** caries prevalence, children, Turkey, meta-analysis, oral health

## Abstract

**Objectives**: This meta-analysis aims to estimate the pooled prevalence of dental caries among children in Turkey by synthesizing national evidence from theses and peer-reviewed articles published between 2000 and 2024. **Methods**: Studies were retrieved from the YÖK Thesis Database and Google Scholar using keywords such as ‘Caries Prevalence’ and ‘Primary Tooth.’ PubMed was used to cross-check peer-reviewed articles. A total of 28 studies and 29 data points including 40,244 children aged approximately 2 to 15 years were included. PRISMA guidelines were followed. Heterogeneity was assessed using the I^2^ index, and publication bias was evaluated with an Egger’s test and funnel plots. **Results**: The pooled prevalence of dental caries among Turkish children was 75.6% (95% CI: 70.8–79.8%). Substantial heterogeneity was observed (I^2^ = 98.4%), but no significant publication bias was detected. **Conclusions**: Dental caries remains a significant public health issue among children in Turkey. Targeted preventive measures—such as nationwide dental screenings and school-based fluoride programs—are urgently needed.

## 1. Introduction

Dental caries is one of the most common chronic diseases worldwide and continues to be a significant public health concern in pediatric populations [1,2]. Dental caries can be defined as a biofilm-mediated dysbiosis that involves changes in the microbiome composition and function, which leads to the demineralization of tooth tissues due to the fermentation of dietary carbohydrates, producing acid by select oral bacteria [3].

The burden of dental caries is particularly severe in developing countries, where socioeconomic inequalities, poor oral hygiene practices, limited fluoride exposure, and insufficient access to dental care persist [4,5]. In children, caries is associated not only with pain and infection but also with school absenteeism, impaired physical development, and diminished quality of life [4].

Global prevalence estimates reveal considerable variation across countries and regions. For example, dental caries affects approximately 52% of U.S. children aged 5–9 [1], while a meta-analysis by Kazeminia et al. (2020) [6] reported a global prevalence of 46.2% among both primary and permanent teeth. The Global Burden of Disease Study also emphasizes that untreated dental caries in primary teeth is among the most prevalent health conditions globally, particularly affecting children [7].

Similarly, neighboring countries also report alarmingly high rates of dental caries in children, highlighting a broader regional public health challenge. For example, in Varna, Bulgaria, the caries rate among 5–7-year-olds was 93% [8]. In Iran, 53.2% of children aged 1–5 were found to have early childhood caries (ECC) [9], while in Romania, caries prevalence among 6–8-year-olds ranged from 85.2% to 95.5% [10]. These figures highlight the widespread burden of disease in the region and reinforce the need for coordinated national-level interventions in Turkey.

In contrast, regional studies from Turkey report significantly higher prevalence rates, often exceeding 60–80% [11,12,13]. This suggests that Turkish children may be disproportionately affected, yet no previous study has systematically synthesized national data on this issue.

Despite the availability of numerous regional studies, Turkey still lacks a unified national estimate of dental caries prevalence in children. While high prevalence rates have been reported in several regions—including both urban centers such as Istanbul (the 1st most developed city), Ankara (the capital city), and Izmir (the 3rd most developed city) as well as less developed provinces [13,14,15,16]—there is no centralized system for monitoring geographic disparities. The absence of standardized national data limits the capacity of policymakers to design coherent and equitable oral health interventions. This meta-analysis aims to fill this gap by synthesizing evidence from peer-reviewed publications and academic theses to generate a comprehensive nationwide estimate.

Accordingly, this meta-analysis aims to provide the first nationally pooled estimate of dental caries prevalence among Turkish children. By synthesizing data from both peer-reviewed publications and academic theses published between 2000 and 2024, this study addresses a critical gap in the literature. The findings are expected to inform national oral health planning and support evidence-based preventive strategies, especially in underserved regions with high disease burden.

## 2. Materials and Methods

This meta-analysis was conducted in accordance with the PRISMA (Preferred Reporting Items for Systematic Reviews and Meta-Analyses) guidelines [17]. The primary objective was to estimate the pooled prevalence of dental caries in children residing in Turkey, using data from studies published between January 2000 and October 2024.

### 2.1. Search Strategy and Data Sources

A comprehensive literature search was performed using two primary databases: the YÖK Thesis Database and Google Scholar. Additionally, PubMed was used to verify and cross-check the inclusion of relevant peer-reviewed articles, but it was not part of the primary search. Search terms included combinations of the following keywords in both Turkish and English: “Caries Prevalence,” “Primary Tooth,” “Dental Caries,” and “Children.” Boolean operators (AND/OR) were applied to maximize sensitivity. Although Google Scholar is not typically recommended for systematic searches due to its lack of reproducibility and advanced filtering options, it was included to access Turkish academic theses and gray literature not available in international databases. The complete list of search terms and combinations is provided in Supplementary Table S1.

### 2.2. Eligibility Criteria

Studies were included if they

- Focused on children aged 2–15 years residing in Turkey;
- Reported the prevalence of dental caries (either in primary, permanent, or both types of teeth);
- Were based on cross-sectional designs;
- Provided full-text access.

Studies were excluded if they

- Were review articles, case-control or cohort studies, or case reports;
- Used alternative caries metrics (e.g., active/severe caries);
- Studies that used DMFT (Decayed, Missing, and Filled Teeth) scores as the sole outcome were excluded if they did not provide caries prevalence as a percentage;
- Had unclear methodologies or did not specify diagnostic criteria;
- Were inaccessible in full text.

### 2.3. Study Selection

A total of 1322 records were initially retrieved (1319 from the two databases and 3 from manual searches). After removal of duplicates and screening of titles and abstracts, 189 studies were retained for full-text review. Of these, 28 studies met the inclusion criteria. One of these studies reported separate prevalence estimates for two distinct age groups (14 and 15 years), resulting in a total of 29 data points used in the meta-analysis.

Study selection was performed by a single reviewer due to time and resource constraints. Although dual screening is generally recommended to enhance reliability, this limitation has been acknowledged and transparently discussed in the manuscript.

### 2.4. Data Extraction

Data were extracted into a structured Excel sheet, including the following:

- Author(s) and publication year;
- Sample size and average age;
- Study location;
- Reported caries prevalence;
- A standardized checklist was used to ensure consistency across data points.

Subgroup analysis by age group, geographic region, or dentition type was not feasible due to inconsistent reporting of such variables across the included studies.

Although diagnostic methods were reviewed during data extraction, a significant number of studies did not explicitly specify the criteria used to assess caries (e.g., WHO guidelines, ICDAS, or visual-tactile methods). Therefore, a consistent classification of diagnostic approaches could not be applied across all included studies.

### 2.5. Quality Assessment

A formal risk of bias assessment was not performed due to the descriptive nature of prevalence studies and the heterogeneity of sources (theses vs. peer-reviewed articles). However, only studies with clear methodology and full-text access were included to minimize potential bias.

### 2.6. Ethical Considerations

As this study involved secondary data from published sources, no ethical approval was required.

### 2.7. Statistical Analysis

Meta-analytical computations were conducted using Comprehensive Meta-Analysis (CMA) software, Version 3.3.070. Heterogeneity was assessed using Cochran’s Q test and the I^2^ statistic. A random-effects model was applied due to expected methodological and population-level variations across studies. Publication bias was evaluated using Egger’s regression intercept, Kendall’s tau, and Orwin’s fail-safe N. Funnel plots were also visually examined.

Statistical significance was set at *p* < 0.05 for all tests. The primary goal of this meta-analysis was to evaluate the prevalence of dental caries in children.

## 3. Results

### 3.1. Study Inclusion and Final Sample

**Figure 1:**
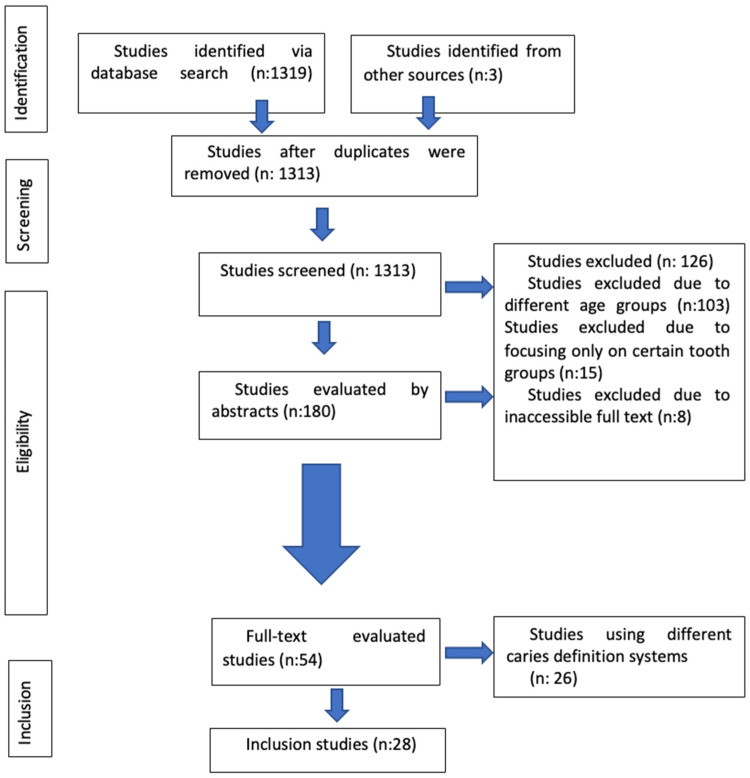
PRISMA flow diagram.

A total of 1322 records were initially retrieved through database and manual searches. After removing duplicates, 1313 studies remained. Title and abstract screening led to the exclusion of 1133 records unrelated to the study topic. Among the remaining 180 studies, 8 were excluded due to lack of full-text access, 103 were excluded due to incompatible age groups, and 15 were excluded for focusing on specific tooth groups or using nonstandard definitions of caries. Ultimately, 28 studies and 29 data points were included in the meta-analysis (Figure 1, PRISMA flow diagram).

### 3.2. Study Characteristics

The included studies encompassed a total of 40,244 children, with sample sizes ranging from 39 to 13,836 participants. The mean age of participants varied across studies, generally ranging from 2 to 15 years. All studies assessed the prevalence of dental caries, and none relied solely on severity indices or limited tooth groups.

The prevalence of dental caries reported in the included studies showed considerable variability, ranging from 34.8% [18] to 94.5% [14]. Most studies reported prevalence rates above 60%, indicating a widespread burden of disease across different age groups and regions (See Table 1 for detailed study-level data).

In studies with larger sample sizes conducted by Ozer et al. [19] with 4341 participants and Dogan et al. [20] with 13,836 participants, prevalence rates of 81.2% and 69.6% were reported, respectively.

In contrast, smaller-scale studies tended to yield more variable results, often reflecting specific subpopulations or localized conditions (See Table 1 for detailed study-level data).

Geographically, data were collected from a wide range of cities including Ankara (the capital city), Istanbul (the 1st most developed city), Izmir (the 3rd most developed city), Trabzon (a major city in the Black Sea region), Kayseri (an industrial and cultural center in Central Anatolia), and Malatya (a key city in Eastern Anatolia), among others. However, due to inconsistent regional labeling and reporting, a detailed regional subgroup analysis could not be performed (See Table 1 for detailed study-level data).

### 3.3. Pooled Prevalence of Dental Caries

The overall pooled prevalence of dental caries among Turkish children was estimated at 75.6% (95% CI: 70.8–79.8%, *p* < 0.001), based on a random-effects model due to high heterogeneity (Table 2).

**Figure 2:**
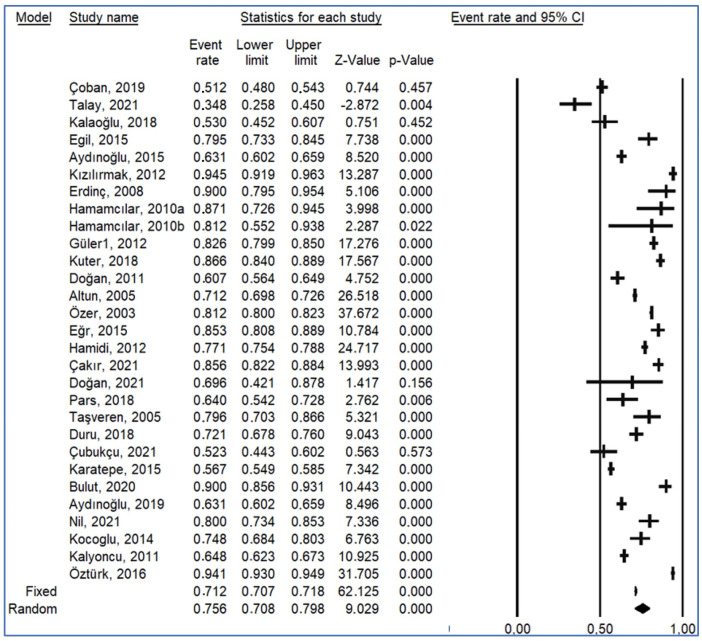
Forest plot of caries rates [11,12,13,14,15,16,18,19,20,21,22,23,24,25,26,27,28,29,30,31,32,33,34,35,36,37,38,39].

The forest plot (Figure 2) illustrates the variability in prevalence estimates across studies. The wide confidence interval suggests substantial heterogeneity in study outcomes, further supporting the use of a random-effects model.

When grouped by sample size, smaller-scale studies (n < 200) often reported higher caries rates (e.g., >80%) compared to larger studies, which tended to yield more moderate prevalence values.

### 3.4. Heterogeneity

There was substantial heterogeneity among the included studies (Q = 1760.44, *p* < 0.001; I^2^ = 98.4%), as reported in Table 2, indicating considerable variation across study findings. This justified the use of the random-effects model.

This heterogeneity may be attributed to differences in diagnostic criteria (e.g., WHO, ICDAS), sample characteristics, age group distributions, and geographic coverage across the studies.

To evaluate the robustness of the pooled prevalence estimate, a sensitivity analysis was conducted by sequentially excluding each study. The pooled prevalence remained stable, ranging between 74.9% and 76.1%, and the confidence intervals showed no significant deviation. These results suggest that no single study disproportionately influenced the overall findings.

### 3.5. Publication Bias

Publication bias was assessed using Orwin’s fail-safe N, Egger’s regression intercept, and Kendall’s tau:

Orwin’s fail-safe N was calculated at 17,888, suggesting that a very large number of unpublished null studies would be required to negate the findings.

Egger’s regression intercept was 2.66 (*p* = 0.142), indicating no significant publication bias.

Kendall’s tau was 0.071 (*p* = 0.293), also indicating non-significant asymmetry.

**Figure 3:**
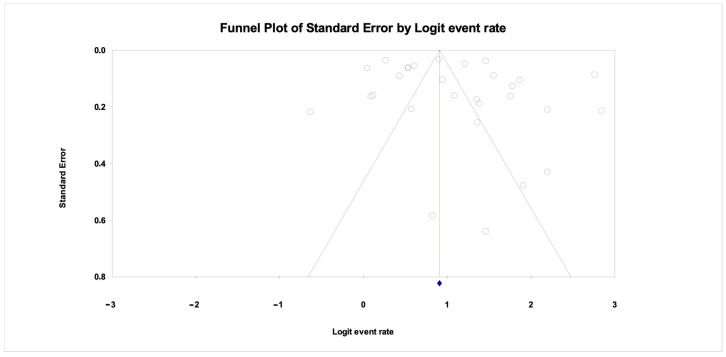
Funnel Plot. (Each grey circle represents an individual study included in the meta-analysis. The diamond at the bottom indicates the pooled effect estimate).

The funnel plot (Figure 3) showed a generally symmetric distribution.

Together, these findings suggest that the pooled prevalence estimate is unlikely to be affected by significant publication bias.

## 4. Discussion

Dental caries remains a widespread chronic condition affecting children globally, with prevalence rates particularly high in developing countries due to socioeconomic disparities, poor oral hygiene, and limited access to preventive care [6]. In Turkey, regional studies have shown prevalence rates exceeding 60–80% [11], a trend confirmed by the current meta-analysis estimating a pooled prevalence of 75.6% among children.

These findings are in line with regional data from neighboring countries. For instance, caries prevalence rates range from 85.2% to 95.5% among 6–8-year-old children in Romania and reach 93% in Bulgarian children aged 5–7. Similarly, early childhood caries prevalence in Iran has been reported at 53.2% among children aged 1–5 [8,9,10]. These comparable figures suggest that Turkey’s burden of pediatric dental caries reflects a broader regional challenge.

Although our meta-analysis did not explore causal factors, the high caries burden may be contextualized by prior literature, which has linked poor oral health outcomes to factors such as limited fluoride access, low parental awareness, and weak preventive systems [3]. These challenges are global; the WHO’s 2010 oral health goals remain unmet in many countries [7].

These patterns align with recent global health trends reported in large-scale epidemiological surveys, including the Global Burden of Disease Study [40]. Socioeconomic status, parental education, dietary habits, and preventive dental behaviors are all well-documented predictors of caries prevalence [5]. Evidence also suggests that mothers with untreated caries are more likely to transmit cariogenic bacteria to their children via shared utensils or poor feeding practices [41].

Despite the high heterogeneity (I^2^ = 98.4%), a sensitivity analysis confirmed that the pooled prevalence remained consistent when each study was excluded one at a time. This supports the robustness of our findings and reduces concerns about any single study disproportionately influencing the results. The substantial heterogeneity found in this meta-analysis may stem from variability in diagnostic methods, regional differences in oral health policies, and inconsistent caries measurement criteria across studies. Nonetheless, this heterogeneity underscores the need for standardized oral health surveillance systems.

A descriptive review of the included studies suggests that dental caries prevalence among children in Turkey has remained consistently high over the period 2000–2024. Due to heterogeneity in study design, reporting, and diagnostic criteria, no clear upward or downward trend could be identified across time.

This study represents the first national-level meta-analysis to synthesize caries prevalence data among children in Turkey. Despite its limitations, including language restriction and single-reviewer screening—both of which are discussed in detail below—the study’s strength lies in its large pooled sample size and inclusion of both theses and peer-reviewed studies.

Given the burden of untreated caries and its long-term consequences—including impaired nutrition, school absenteeism, and lowered quality of life—there is a pressing need for targeted public health initiatives. These should include parent-focused oral health education, school-based fluoride programs, and the integration of dental care into pediatric health services.

### Limitations

One limitation of this meta-analysis is that study selection and screening were performed by a single reviewer, which may have introduced selection bias or reduced reproducibility. Future reviews would benefit from dual independent screening to enhance methodological rigor.

The lack of subgroup-level data limited our ability to conduct more detailed stratified analyses, which could have provided additional insights.

In addition, restricting the inclusion criteria to studies published in Turkish may have led to the exclusion of relevant international data and introduced language bias. Furthermore, variations in diagnostic criteria across studies (e.g., WHO standards, ICDAS, and unspecified visual-tactile assessments) may have contributed to heterogeneity in prevalence estimates. In several studies, the diagnostic criteria for caries assessment were unclear or missing, further limiting our ability to standardize subgroup analyses or compare methodologies.

Additionally, the absence of a formal risk of bias assessment, due to the descriptive nature of the included studies, may limit the internal validity of the findings.

## 5. Conclusions

This meta-analysis provides the first nationwide estimate of dental caries prevalence among children in Turkey, revealing a notably high pooled prevalence of 75.6%. These findings confirm that dental caries remains a major public health burden in Turkish pediatric populations.

While causal factors were not directly assessed, the findings highlight the urgent need for comprehensive oral health strategies.

Future efforts should prioritize early intervention and national data standardization, alongside specific, evidence-based measures such as community water fluoridation, school-based sealant programs, and parental oral health education to address this widespread challenge.

## Figures and Tables

**Table 1:** Features of the Studies Included.

Author/Year	Sample Size	Average Age	Study Location	Caries Prevalence (%)
Çakır [11] 2021	500	-	Konya	85.60
Kuter [12] 2018	763	6	İzmir	86.63
Öztürk ve ark. [13] 2016	2358	-	Adıyaman	94.06
Kızılırmak [14] 2012	420	9.48	Ankara	94.50
Özgül Erdinç [15] 2008	60	10.5 ± 2.3	İstanbul	90
Bulut ve ark. [16] 2020	251	4.57 ± 0.78	İzmir	90
Talay [18] 2021	92	8.5 ± 1.5	Ankara	34.78
Özer ve ark. [19] 2003	4341	8.45	Ankara	81.21
Doğan ve ark. [20] 2021	1,3836	7.04 ± 0.37	Kayseri	69.6
Çoban [21] 2019	962	4.3 ± 0.7	Aydın	51.2
Kalaoğlu [22] 2018	157	7.47 ± 1.66	İstanbul	53
Eğil [23] 2015	200	9.34 ± 1.89	İstanbul	79.5
Oğlak Aydınoğlu [24] 2015	1083	2.95 ± 3.60	Trabzon	63.10
Hamamcılar [25] 2010	39	14	Niğde-Yalova	87.1
Hamamcılar [25] 2010	16	15	Niğde-Yalova	81.2
Güler ve ark. [26] 2012	856	11.42 ± 1.86	Malatya	82.60
Doğan ve ark. [27] 2011	499	34.8 ±11.23	Karaman-Kütahya	60.72
Altun ve ark. [28] 2005	4186	7.47	Ankara	71.2
Eğri ve ark. [29] 2015	300	12	Tokat	85.3
Hamidi ve ark. [30] 2012	2348	8068 ± 0360	Kırklareli	77.10
PARS ve ark. [31] 2018	100	4.39 ± 0.9	Ankara	64.00
Kambek Taşveren ve ark. [32] 2015	94	12	Sivas	79.61
Duru ve ark. [33] 2018	451	8.54 ± 1.13	Eskişehir	72.1
Elbek Çubukçu [34] 2021	150	4.7 ± 0.5	Bursa	52.3
Karatepe ve ark. [35] 2015	3020	12	Sakarya	56.70
Aydınoğlu [36] 2019	1077	-	Trabzon	63.1
Yüksekkaya [37] 2021	175	52.99 ± 8.44	İstanbul	80.0
Kocoğlu ve ark. [38] 2014	205	9.06 ± 1.8	Konya	74.8
Kalyoncu ve ark. [39] 2011	1405	-	Eskişehir	64.80

**Table 2:** Meta-analysis results.

Model	N	Estimated Values			Heterogeneity Test Results		
		Event Rate (%95 CI *)	Z-Value	*p*	Q	*p*	I^2^
Fixed	29	0.712 (0.707–0.718)	62,125	<0.001	1760.436	<0.001	98.409
Random		0.756 (0.708–0.798)	9029	<0.001			

* CI: Confidence Interval.

## Data Availability

The data presented in this study are available on request from the corresponding author due to ethical considerations and institutional restrictions regarding the redistribution of thesis-based and previously published data.

## Supplementary Materials

The following supporting information can be downloaded at: https://www.mdpi.com/article/10.3390/children12060777/s1, Table S1: PRISMA 2020 Checklist [42].

## Abbreviations

The following abbreviations are used in this manuscript:

PRISMA	Preferred Reporting Items for Systematic Reviews and Meta-Analyses
YÖK	Higher Education Council
DMTF	Decayed, Missing, and Filled Teeth
